# Application of Live-Cell RNA Imaging Techniques to the Study of Retroviral RNA Trafficking

**DOI:** 10.3390/v4060963

**Published:** 2012-06-08

**Authors:** Darrin V. Bann, Leslie J. Parent

**Affiliations:** 1 Penn State College of Medicine, Department of Medicine, 500 University Dr., Hershey, PA 17033, USA; Email: dbann@hmc.psu.edu; 2 Penn State College of Medicine, Department of Microbiology & Immunology, 500 University Dr., Hershey, PA 17033, USA

**Keywords:** RNA labeling, retrovirus, live-cell

## Abstract

Retroviruses produce full-length RNA that serves both as a genomic RNA (gRNA), which is encapsidated into virus particles, and as an mRNA, which directs the synthesis of viral structural proteins. However, we are only beginning to understand the cellular and viral factors that influence trafficking of retroviral RNA and the selection of the RNA for encapsidation or translation. Live cell imaging studies of retroviral RNA trafficking have provided important insight into many aspects of the retrovirus life cycle including transcription dynamics, nuclear export of viral RNA, translational regulation, membrane targeting, and condensation of the gRNA during virion assembly. Here, we review cutting-edge techniques to visualize single RNA molecules in live cells and discuss the application of these systems to studying retroviral RNA trafficking.

## 1. Introduction

Recombinant green fluorescent protein (GFP) was first cloned over 25 years ago, permitting fluorescent labeling of proteins for *in vivo* trafficking and localization studies [1,2]. Until recently, there has been no analogous system to visualize RNA trafficking in living cells, so many RNA localization studies have used fluorescently-labeled probes to visualize RNA transcripts in fixed cells. However, these studies only provide a snapshot of RNA localization at a single point in time. On the other hand, live cell RNA tracking studies allow the dissection of each step in RNA metabolism, including transcription, post-transcriptional processing, nuclear export, post-transcriptional regulation, and RNA decay. Accordingly, over the last decade there has been a rapid expansion of methods to visualize single RNA molecules in living cells.

The first experiments to visualize the localization of mRNA molecules in live cells involved microinjection of fluorescently labeled full-length mRNAs [3]. However, this approach is time consuming, technically challenging, and may not reflect the trafficking pathways of native transcripts. Techniques used to visualize endogenous RNA rely on the binding of fluorescent oligonucleotides, small molecules, or protein reporters to a specific sequence within the transcript of interest. Using fluorescent molecules to visualize RNA in living cells is limited by the need to deliver the reporter without causing cellular toxicity. This problem can largely be overcome by using sequence-specific RNA binding proteins labeled with a fluorophore such as GFP and titrating expression levels. Furthermore, most RNA tracking systems have been modified or adapted to increase the signal-to-noise ratio, allowing the detection of single RNA molecules over background fluorescence from unbound reporter. As a result, these systems have provided valuable insight into the localization and regulation of cellular RNAs. 

In addition to tracking cellular mRNAs, fluorescent RNA labeling techniques are also well suited to study viral RNA trafficking. RNA synthesis is essential for the replication of all viruses, however cellular defensive strategies have evolved to recognize and destroy viral RNA. As a result, viruses have developed diverse mechanisms to facilitate viral replication while circumventing host antiviral defenses. Many, if not all, RNA viruses interact with cellular RNA processing machinery to disable cellular defenses against viral infection, or to facilitate viral replication. Therefore, studying the trafficking of viral RNA in living cells may provide insight into how cells defend against viral invasion and how viruses are able to avoid cellular defenses and facilitate their own replication.

Retroviruses are positive stranded RNA viruses characterized by the ability to reverse-transcribe their RNA genomes into DNA and stably integrate into the chromosome. Following integration all retroviruses express full-length RNA that fulfills two roles in the viral replication cycle: (i) to serve as a viral mRNA to direct the synthesis of the retroviral structural protein, Gag; and (ii) to function as a genomic RNA (gRNA), which is encapsidated into nascent virus particles. Genome encapsidation is initiated when the gRNA is bound by Gag, however for most retroviruses this process occurs in *trans*, meaning that Gag does not bind the RNA from which it was translated [4]. This feature of retroviral replication suggests that there may be a mechanism to spatially separate viral RNAs destined for encapsidation from those to be used for translation. Accordingly, tracking the intracellular fate of full-length retroviral RNAs can provide insight into retroviral RNA trafficking and can serve as a model system to study post-transcriptional regulation of cellular mRNAs. Here, we review some of the most common methods used to visualize single RNA molecules in living cells with a focus on how these techniques can be applied to the study of retroviral RNA trafficking.

## 2. Hybridization-Based RNA Labeling Techniques

Radiolabeled RNA probes were first used to study ribosomal gene amplification in *Xenopus* in 1969 [5]. Although the technology available to image specific nucleic acid sequences has improved dramatically over the last 50 years, hybridization-based approaches still use the same principles to visualize RNA in cells. In each of these approaches, fluorescently labeled oligonucleotide probes complimentary to the transcript of interest are introduced into live cells. Single molecule sensitivity can be achieved by using several probes targeting the same transcript, or by reducing background signal associated with unbound probe.

### 2.1. Fluorescence *in situ* Hybridization (FISH)

The purpose of this article is to review techniques used to visualize RNA localization in living cells, however FISH deserves a brief treatment here. Like all hybridization-based techniques, FISH detects endogenous or engineered RNA (or DNA) molecules using fluorescently-labeled probes complimentary to the sequence of interest. Two approaches used to achieve the signal-to-noise ratio required for single mRNA visualization include the use of 4–10 multiply-labeled probes [20], or using many (40+) singly-labeled probes [21]. Each of these approaches offers relative advantages and disadvantages in terms of sensitivity, specificity, and quantitation; however they share common drawbacks. The first limitation is that FISH requires samples to be fixed and therefore does not provide dynamic information about RNA trafficking or transient interactions with host factors. Additionally, fixation itself can affect signal intensity and may disrupt the integrity of certain organelles [22]. Furthermore, non-hybridized probe can remain within the sample, reducing the signal-to-noise ratio. Despite these limitations, single molecule FISH can provide quantitative data regarding the expression and localization of endogenous transcripts, which is advantageous over some of the other systems discussed here.

### 2.2. Linear Oligonucleotide Probes

Similarly to FISH, RNAs can be visualized in living cells using fluorescently-labeled, linear probes complementary to the sequence of interest (Table 1) [23]. However, in live cells, unbound probe cannot be removed by washing. As a result, this approach requires the introduction of multiple unique probes targeting the same transcript so that localized concentrations of probes can be differentiated from high levels of background fluorescence [7]. Linear probes have been useful in visualizing RNAs associated with small nuclear ribonucleoprotein complexes (snRNPs) and ribosomal RNAs associated with nucleoli [6,7]. However, limitations including low signal-to-noise ratios, the need to microinject probes into cells of interest, and the tendency of probes to rapidly accumulate in the nucleus following microinjection have discouraged more widespread use of this approach [22].

To increase the signal-to-noise ratio associated with linear probes, some groups have utilized Förster resonance energy transfer (FRET)-based systems [8,9]. In this approach, two probes complementary to adjacent regions on the transcript of interest are labeled with donor and acceptor fluorophores, respectively. Hybridization of each probe to the transcript brings the fluorophores into close proximity, resulting in FRET when the donor fluorophore is excited (Table 1) [8]. Both probes must bind the transcript of interest to produce a FRET signal, increasing the specificity of this system over single linear probes [8]. The use of FRET also increases the signal-to-noise ratio of the system, because FRET only occurs when both probes are bound to the same transcript. The FRET signal can be further increased through the addition of multiple donor fluorophores [24]. Alternatively, background FRET signal can be reduced through the use of autoligation FRET probes. In this system, the donor probe is labeled with a fluorophore and a quencher, preventing fluorescence of the donor. Hybridization of the donor and acceptor probes to the transcript results in autoligation of the donor and acceptor probes with excision of the quencher, producing a FRET signal (Table 1) [10,11]. While FRET-based systems may overcome some of the sensitivity issues associated with single linear probes, they share the same delivery obstacles. As a result, these systems have not been used extensively to visualize viral RNA in living cells.

**Table 1:** Methods to visualize RNA in living cells.

Method	Diagram	Recognition Sequence	Citations
Linear Oligonucleotide Probe	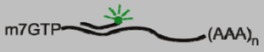	User defined	[6,7]
Linear FRET Probe	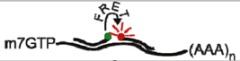	User defined	[8,9]
Autoligation FRET Probe	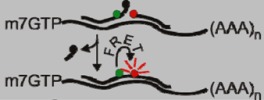	User defined	[10,11]
Molecular Beacon	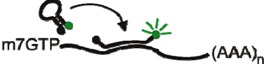	User defined	[12]
MS2-GFP	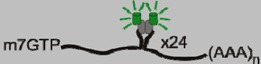	19 nucleotide stem-loop	[13,14]
Bgl-mCherry	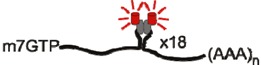	29 nucleotide stem-loop	[15]
λ_N_-GFP	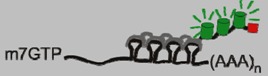	15 nucleotide stem-loop	[16]
PUM-HD	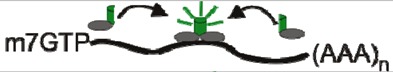	User defined	[17,18]
Spinach	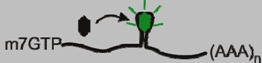	Varies based on desired fluorescence	[19]

### 2.3 Molecular Beacons

Unlike linear probes, molecular beacons form stem-loop structures. The 5’ and 3’ ends of the beacon form a self-complimentary stem, while the central loop region is complimentary to the transcript of interest [12]. The 5’ and 3’ ends of the molecule are conjugated to a fluorophore and a quencher, which prevents fluorescence when the beacon is in a stem-loop conformation. Binding of the beacon to the transcript induces melting of the molecular beacon stem structure, moving the fluorophore away from the quencher and resulting in fluorescence (Table 1). A further increase in the signal-to-noise ratio associated with molecular beacons can be achieved using FRET between two molecular beacons labeled with donor and acceptor fluorophores [25]. Owing to the decreased background fluorescence associated with molecular beacons compared to linear probes, molecular beacons have been used more extensively to visualize viral RNAs in living cells. This approach has been used to monitor the spread of bovine and human respiratory syncitial viruses [26,27] and coxsackievirus B6 [28] in tissue culture. Additionally, molecular beacons have been used to study the intracellular trafficking dynamics of poliovirus positive-stranded RNA [29] and influenza A virus mRNA [30].

To study viral RNA trafficking, molecular beacons offer some advantages over the fluorescent protein-based RNA labeling techniques discussed below. Primarily, because molecular beacons hybridize to a sequence of interest, this approach can be used to visualize native (*i.e.*, non-engineered) viral RNAs. As a result, molecular beacons can be used to visualize viral RNAs in clinical isolates [26], obviating the need to introduce a foreign RNA sequence into the viral RNA of interest. However, the use of molecular beacons to study retroviral RNA trafficking may be complicated by the presence of endogenous proviruses, which frequently share a high degree of sequence homology with exogenous retroviruses. For example, many strains of laboratory mice express up to eight endogenous retroviruses with high homology to exogenous mouse mammary tumor virus (MMTV) [31]. Consequently, care must be taken to design molecular beacons that effectively distinguish exogenous retroviral RNA from RNA expressed by endogenous retroviruses.

An additional obstacle to the use of hybridization-based probes, including molecular beacons, is the delivery of probes into living cells. Microinjection is the most direct delivery method and allows for very rapid visualization of target RNAs [32], but poses technical challenges. Accordingly, other methods are frequently used to deliver molecular beacons into cells. One approach is to reversibly permeabilize cells using streptolysin O [26,30]. This technique offers rapid visualization of target RNAs without the technical requirements of microinjection [23]. Rapid introduction of oligonucleotide probes or molecular beacons into cells can also be accomplished by conjugating the probe to a cell-penetrating peptide, including HSV-1 VP22 or HIV-1 Tat [22]. Alternatively, cationic lipid-based transfection reagents have been used to deliver molecular beacons into cells [29]. Unlike permeabilization or cell-penetrating peptides, however, these reagents may deliver molecular beacons to the endocytic pathway, resulting in nuclease degradation of the beacon and increased background fluorescence [22]. However, non-specific fluorescence associated with molecular beacon degradation can be reduced using 2’O-methylated probes [7] or by using specific quantum dot-molecular beacon conjugates [33]. Other transfection methods such as electroporation have also been used to introduce molecular beacons into live cells, though all transfection-based delivery methods impart a lengthy delay before target transcripts can be visualized [22]. As a result, molecular beacons and other hybridization-based techniques can be powerful tools to track viral RNAs in living cells, but all of these approaches share some common limitations and are not universally applicable to every model system.

## 3. RNA Binding Protein-Based RNA Labeling Techniques

The use of oligonucleotide probes presents many challenges to live-cell imaging of single RNA molecules. To circumvent some of these problems, plasmid-based systems have been developed using fluorophore-labeled RNA binding proteins that specifically recognize RNA sequences inserted into the transcript of interest. Alternatively, fluorophore-labeled cellular RNA-binding proteins can be mutated to recognize specific RNA sequences, permitting the visualization of endogenous transcripts.

### 3.1. MS2-GFP

The MS2 RNA-binding protein forms the viral capsid of the MS2 bacteriophage. During the late phase of phage replication, an MS2 dimer binds a 19-nucletide stem-loop structure on the phage genomic RNA, which initiates phage capsid assembly and encapsidation of the genome [34]. In 1998, Bertrand *et al*. [13] took advantage of this system to visualize RNA localization by linking GFP to an MS2 mutant that binds RNA but is incompetent for capsid assembly [35]. A nuclear localization signal (NLS) was added to the MS2-GFP fusion protein to reduce background fluorescence in the cytoplasm. To visualize RNA localization in yeast, six MS2 stem-loops were inserted between the *LacZ* coding region and the *ASH1* 3’ untranslated region (*LacZ-MS2-ASH1*) (Table 1) [13]. When MS2-GFP was expressed alone, the fluorescent signal accumulated in the nucleus by virtue of the NLS. However, when MS2-GFP was coexpressed with *LacZ-MS2-ASH1*, discrete MS2-GFP foci were visualized in the nucleus and cytoplasm. Subsequent refinements demonstrated that inserting 24 copies of the MS2 stem-loop sequence into the RNA yielded single-molecule sensitivity [14]. Interestingly, on average only 33 molecules of GFP were associated with each RNA molecule [14]. Because MS2 binds as a dimer, this result suggests that on average only about two-thirds of the 24 MS2 stem-loops were occupied by MS2-GFP. In fact, Fusco *et al*. [14] visualized single RNA particles containing as few as 20 GFP molecules, indicating that single RNAs can be visualized using smaller numbers of fluorophores.

The use of MS2-GFP offers several advantages to hybridization-based approaches; most notably RNA can be tracked in living cells, obviating the concern for fixation artifacts. Additionally, the high affinity interaction between the MS2 coat protein and the MS2 RNA (Kd = 0.4 nM) results in specific labeling of RNAs containing the MS2 stem loops [36] and the MS2-GFP NLS allows newly synthesized RNA to be bound by the reporter co-transcriptionally [37,38]. Together, these features result in a high signal-to-noise ratio in the cytoplasm [14]. This system does, however, have the disadvantage that MS2-GFP only binds to RNAs engineered to contain MS2 stem-loops, so it cannot be used to track native RNAs. Furthermore, achieving an optimal ratio of MS2-GFP to RNA expression may require titrating different amounts of each expression construct [13]. The localization of free MS2-GFP in the nucleus may partially obscure visualization of nuclear RNAs, although the punctate intranuclear MS2-GFP signal can be brought out by carefully titrating MS2-GFP expression [37,38,39]. Another potential concern arises from the possibility that the RNA-bound MS2-GFP protein could alter normal RNA trafficking. However, RNAs bound by MS2-GFP are efficiently exported from the nucleus and properly localized in the cytoplasm [14,40,41]. Furthermore, retroviral RNAs bound by MS2-GFP are efficiently encapsidated into virus particles [15]. Together, these data suggest that MS2-GFP does not interfere with proper RNA trafficking or binding by the retroviral Gag protein.

Due to its ease of use and relatively few drawbacks, the MS2-GFP system has been used to study retrovirus RNA biology in living cells. Boireau and colleagues [38] took advantage of the high affinity interaction between MS2-GFP and HIV-1 RNAs containing 24 copies of the MS2 stem-loop to study the kinetics of HIV-1 RNA transcription using fluorescence recovery after photobleaching (FRAP). MS2-GFP at sites of HIV-1 transcription was subjected to photobleaching, and fluorescence recovery occurred when free MS2-GFP proteins bound to newly transcribed RNAs bearing the MS2 stem-loops [38]. This allowed the authors to measure the rate of HIV-1 RNA transcription. In addition, due to very slow exchange of RNA-bound MS2-GFP with free MS2-GFP, this system can be used to track single RNA molecules over time. By using a photoactivatable form of GFP, RNA molecules can be differentially labeled at sites of transcription [37]. In theory, this approach could allow the tracking of single RNA molecules throughout the entire process of retrovirus particle formation.

The MS2-GFP system has also provided valuable insight into the viral and cellular factors that regulate the trafficking of retroviral RNAs. The canonical view of retroviral assembly holds that for C-type retroviruses, Gag and the gRNA interact at the plasma membrane to assemble new virus particles. However, using MS2-GFP, Gag-RNA interactions have been visualized at earlier locations in the assembly pathway. For example, the feline immunodeficiency virus (FIV) Gag protein co-localizes with the gRNA on the cytoplasmic face of the nuclear envelope [42], whereas HIV-1 and murine leukemia virus (MLV) Gag-gRNA interactions were visualized on the cytoplasmic face of endosomes [42,43]. The high sensitivity of the MS2-GFP system has also permitted a detailed kinetic analysis of individual HIV-1 virus particle formation at the plasma membrane [44]. Together, these data have provided insight into the intracellular trafficking of retroviral RNAs and the mechanism underlying the incorporation of RNA genomes into virus particles.

Another informative application of the MS2-GFP system is to visualize protein-RNA and RNA-mediated protein-protein interactions in living cells. This approach, called trimolecular fluorescence complementation (TriFC), utilizes MS2 conjugated to a non-fluorescent N-terminal fragment of Venus fluorescent protein (MS2-VN). Binding of an MS2 stem-loop containing RNA by MS2-VN and a target protein containing the C-terminal fragment of Venus (VC) results in reconstitution of the Venus fluorophore and detectable fluorescent signal [45]. Recently, Milev *et al*. [46] used TriFC to probe Gag-Gag and Gag-Staufen interactions on viral RNA. As expected, Gag-VC complemented MS2-VN on *psi*-containing RNAs, however Staufen-VC failed to complement MS2-VN in the absence of Gag [46]. To examine whether Gag could recruit Staufen to viral RNAs, the authors took advantage of the fact that the MS2 protein binds to RNA as a dimer. By co-expressing MS2-Gag with MS2-VN and VC-labeled target proteins, they found that HIV-1 Gag actively recruits Staufen to the viral RNA [46]. This study demonstrates that MS2-GFP is a powerful system to study the dynamic trafficking of viral RNAs and to probe host-virus interactions in living cells.

### 3.2. Bgl-mCherry

The Bgl-mCherry RNA labeling system, analogous to the MS2-GFP system, is derived from the *E. coli* anti-termination protein BglG. The first 58 amino acids comprise the BglG RNA-binding domain, and this protein fragment is sufficient for anti-termination activity [47]. To create a live-cell RNA labeling system, Chen *et al*. [15] fused the N-terminal 52 amino acids of the BglG RNA-binding domain to mCherry and an NLS to create Bgl-mCherry. Bgl-mCherry binds a 29-nucleotide stem-loop structure, and single molecule sensitivity has been obtained using only 18 Bgl stem-loops (Table 1) [15]. However, it is not clear whether all 18 stem-loops are required to achieve this level of sensitivity.

This system shares many of the advantages and disadvantages of the MS2 RNA labeling system. Bgl-mCherry binds with high affinity to RNAs containing BglG stem-loops, allowing selective labeling of the target RNA [15]. However, like MS2, the multiple, highly repetitive stem loop sequences can be unstable, although this problem can be managed by using recombination-deficient bacterial strains. The Bgl-mCherry system has been used successfully to label HIV-1 RNAs, and viral genomes bound by Bgl-mCherry are incorporated into retrovirus particles with >90% efficiency, suggesting that Bgl-mCherry is unlikely to adversely affect viral RNA trafficking [15].

Due to the highly specific binding of MS2-GFP and Bgl-mCherry to their respective RNA sequences, separately labeled RNAs can be discriminated within the same cell [15,48]. Moreover, because MS2-GFP and Bgl-mCherry are efficiently encapsidated into retrovirus particles, the genomic RNA content of single virions can be compared [15,40,48,49]. The Hu laboratory has used this system to study genome encapsidation by HIV-1. Because retroviral genomic RNAs (gRNAs) are encapsidated as a non-covalent dimer, packaging of two heterologous RNAs can lead to recombination during reverse transcription [50]. When *psi-*containing HIV-1 RNAs labeled with MS2-GFP or Bgl-mCherry were co-expressed, the genomic RNAs were randomly assorted into virus particles with the expected 1:2:1 ratio [15]. However, mutations within the genomic RNA dimerization initiation sequence disrupt this ratio, suggesting that dimerization occurs prior to encapsidation [15]. Using the same system, the Hu laboratory also demonstrated that HIV-1 genome dimerization appears to occur in the cytoplasm and that the RNA nuclear export pathway influences the assortment of genomic RNAs virus particles [40]. 

The Bgl-mCherry system has also been used to study the mechanism of encapsidation of genomic RNA by HIV-2 [49] and to investigate cross-encapsidation of HIV-2 RNA by HIV-1 Gag [48]. Other retroviruses, including MMTV and Mason-Pfizer Monkey Virus (MPMV), can also cross-package the genomes of orthologous retroviruses [51] and exogenous retroviruses can recombine with endogenous retroviruses to create novel pathogens [52]. As a result, single particle RNA analysis using MS2-GFP and Bgl-mCherry may provide valuable insight into the frequency and mechanisms of cross-packaging events, and may shed light onto the mechanisms used by different retroviruses to select genomic RNAs for packaging.

### 3.3. λ_N_-GFP

One of the difficulties associated with the MS2-GFP and Bgl-mCherry systems is the insertion of long (e.g., ~1,300 nucleotides for 24 MS2 stem-loops), highly repetitive sequences into the RNA of interest. To circumvent this problem, Daigle and Ellenberg recently developed the λ_N_-GFP system to study intracellular RNA trafficking in living cells [16]. The λ_N_-GFP reporter protein is composed of four tandem repeats of the N-terminal 22 amino acids of the bacteriophage λ N coat protein (λ_N_) fused to three eGFP molecules and an M9 localization signal. The λ_N_-GFP reporter binds four 15-nucleotide stem-loop structures called BoxB, which are inserted into the target RNA (Table 1) [16]. Each set of four BoxB loops adds only ~80 nucleotides, reducing the amount of exogenous sequence within the study RNA. Like other RNA-binding protein-based tracking systems, λ_N_-GFP binds its target RNA with high affinity and specificity; each λ_N_ repeat binds a single BoxB stem-loop with Kd = 22 nM [53]. As with MS2-GFP and Bgl-mCherry, λ_N_-GFP binding could influence the trafficking of the target RNA molecule. However, actin zipcode-containing mRNAs bound by λ_N_-GFP are properly localized, providing evidence that λ_N_-GFP may not alter the trafficking of mRNAs to which it binds [16].

**Figure 1:**
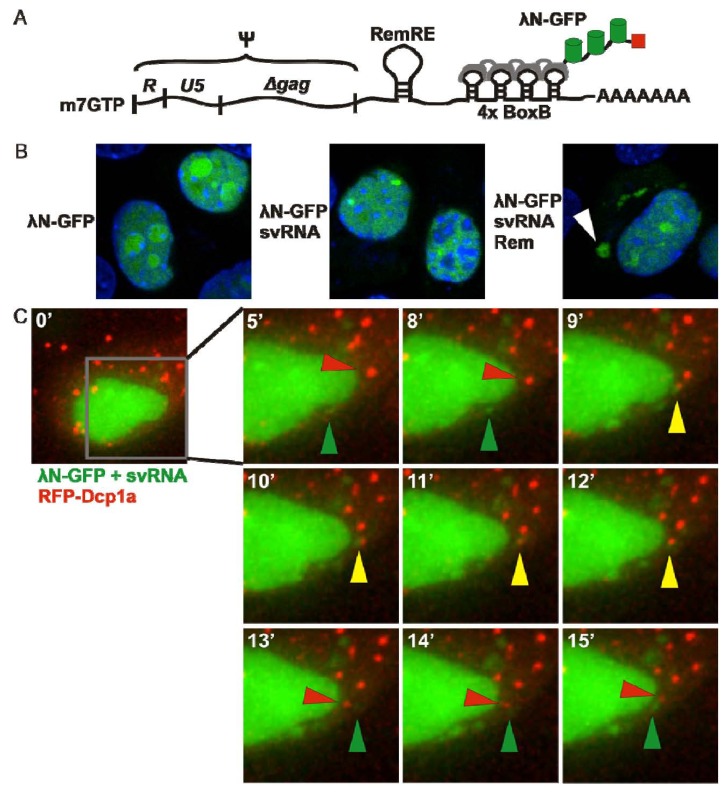
Localization of mouse mammary tumor virus (MMTV) RNA with P-body proteins. (**A**) Diagram of MMTV subviral RNA containing the MMTV packaging signal (Ψ), the Rem response element (RemRE), and 4 BoxB loops bound by the λ_N_-GFP reporter. (**B**) NMuMG cells transfected with λ_N_-GFP alone (left); λ_N_-GFP and svRNA (middle); or λ_N_-GFP, svRNA, and Rem (right). Fixed cells were imaged using a Leica SP2 confocal microscope. White arrow indicates cytoplasmic accumulation of λ_N_-GFP bound to svRNA. (**C**) Live-cell co-imaging of MMTV svRNA and RFP-Dcp1a in NMuMG cells. Green arrows indicate svRNA alone; red arrows indicate RFP-Dcp1a alone; yellow arrows indicate colocalization between the svRNA and RFP-Dcp1a.

To visualize MMTV RNA trafficking in living cells, we created a previously unpublished subviral RNA (svRNA) construct containing the MMTV Ψ packaging sequence [54]; the Rem response element, which is bound by the MMTV Rem protein to export full-length viral RNA from the nucleus [55,56,57]; and four BoxB loops (Figure 1A) [16]. When expressed alone in normal mouse mammary epithelial (NMuMG) cells, λ_N_-GFP was localized to the nucleus as expected (Figure 1B, left). Co-expression of the svRNA with λ_N_-GFP resulted in no detectable cytoplasmic GFP signal (Figure 1B, middle) although co-expression of Rem, svRNA, and λ_N_-GFP resulted in export of λ_N_-GFP from the nucleus, supporting the finding that Rem is required for the nuclear export of MMTV RNA (Figure 1B, right) [56]. Interestingly, the MMTV RNA accumulated in cytoplasmic granules reminiscent of mRNA processing sites known as P-bodies. To address whether MMTV RNA was associated with P-body proteins, NMuMG cells were transfected with svRNA, Rem, λ_N_-GFP, and RFP-Dcp1a, a P-body component [58]. Living cells were imaged on a DeltaVision Elite microscope with a 60X 1.4 NA objective in a 37 °C environmental chamber. A subset of MMTV RNA granules transiently trafficked to P-bodies containing RFP-Dcp1a (Figure 1C). Together, these data demonstrate the feasibility of the λ_N_-GFP system for tracking retroviral RNAs in living cells. 

### 3.4. PUM-HD

One of the major drawbacks to the RNA-binding protein-based systems discussed to this point is that all of these systems require the insertion of specific stem-loop structures into the RNA of interest. On the other hand, although endogenous transcripts can be visualized using the hybridization-based techniques discussed earlier, these techniques are technically challenging. These limitations have resulted in the development of a genetic probe based on the RNA-binding protein PUMILIO1 to track endogenous RNAs in living cells [17]. The PUMILIO1 homology domain (PUM-HD) is composed of eight repeating units, each of which binds a single RNA nucleotide [59,60]. The wild-type PUM-HD recognizes the RNA sequence 5’ UGUA(U/C)AUA, however PUM-HD can be engineered to recognize different sequences by mutating individual units based on a predictable set of rules [61,62].

To visualize endogenous RNA molecules in living cells Ozawa *et al*. created two PUM-HD mutants that recognized adjacent sequences on the mitochondrial NADH dehydrogenase 6 RNA [17]. The PUM-HD constructs were fused to the N- and C-terminal fragments of a split GFP reporter, so GFP fluorescence was only produced when both PUM-HD constructs were bound to the same RNA (Table 1) [17]. Due to the high signal-to-noise ratio achieved through the split GFP reporter, this system has been used to visualize single, endogenous RNAs in living cells [18]. The PUM-HD system has also been used to study the trafficking of tobacco mosaic virus RNA in living plant cells, and RNA localization appears to be unaffected by PUM-HD binding [63]. As a result, the PUM-HD system may be a powerful technique to study trafficking of RNAs expressed from endogenous retroviruses and during authentic retroviral infection.

As with all RNA tracking systems, however, the PUM-HD system has some limitations. Background fluorescence may result from non-specific complementation of the split GFP fluorophore, reducing the signal-to-noise ratio [63]. Also, because PUM-HD recognizes endogenous RNAs, it has the potential to bind off-target RNAs much like an oligonucleotide probe. However, by recognizing two different eight nucleotide sequences in tandem, the PUM-HD system is theoretically able to distinguish its target from 4.3 × 10^9^ different transcripts [17]. Accordingly, PUM-HD probes should provide the specificity required to recognize the target RNA even in the presence of transcripts with similar sequences. As with hybridization-based approaches, proper controls are necessary to ensure that the PUM-HD probes specifically bind the sequence of interest.

## 4. “Spinach” RNA Tracking System

A common concern for all probe- or RNA binding protein-based RNA labeling systems is that binding of the reporter to the transcript of interest has the potential to alter trafficking of the transcript. Accordingly, an ideal system may be one where the RNA itself directly encodes a traceable marker. Recently, Paige *et al*. described a series of RNA aptamers called “Spinach” that fluoresce when bound to GFP-like fluorophores (Table 1) [19]. The cell-permeable fluorophores are non-fluorescent in the absence of Spinach RNA and are non-toxic, making this system useful for live cell imaging [19]. Furthermore, different combinations of RNA aptamers and fluorophores yield fluorescent RNAs with different excitation and emission spectra [19]. Although the RNA-fluorophore interaction is highly specific [19], single molecule sensitivity has not been established in this system. Furthermore, while the Spinach system eliminates concerns regarding the influence of an oligonucleotide or protein reporter on RNA trafficking, the Spinach aptamer itself could alter the trafficking of the transcript. However, Spinach-labeled 5S RNA appropriately relocalized from a diffuse cytoplasmic distribution to stress granules in 293T cells subjected to osmotic stress [19], suggesting that Spinach aptamers may be useful to follow authentic RNA trafficking and localization. Although the Spinach system offers great promise as a novel system to visualize RNA transcripts in living cells, its utility for tracking single RNA molecules remains to be validated.

## 5. Conclusions

Technological and methodological advances have continued to improve the visualization of single RNA molecules in living cells. However, live cell RNA tracking studies are still not routine and technical challenges persist. For example, while hybridiziation-based approaches such as molecular beacons offer high sensitivity, delivery of the probe into cells can be difficult and requires careful optimization [22]. On the other hand, RNA binding-protein based systems are plasmid-encoded and are therefore relatively easy to manipulate and deliver into cells by transfection. However, these approaches require the engineering of expression constructs and may introduce artifacts due to overexpression or mis-targeting of the transcript [64].

Clearly, not all RNA tracking systems are suitable to address every biological question. Molecular beacons and other hybridization-based approaches can be used to visualize endogenous transcripts, and because molecular beacons fluoresce only when bound to the target transcript, they can detect rapid changes in gene transcription [65]. However, high levels of sequence homology among endogenous and exogenous retroviruses may complicate the use of molecular beacons to study retroviral RNA trafficking. Fortunately, it is possible to design molecular beacons that discriminate between transcripts differing by a single nucleotide [66], providing sufficient specificity to track only exogenous retroviral transcripts. As with all hybridization-based approaches, however, proper controls are necessary to ensure selective binding of the probe to the target RNA.

RNA binding protein-based approaches to live cell RNA tracking including MS2-GFP, Bgl-mCherry, and λ_N_-GFP avoid potential pitfalls in differentiating between two closely related transcripts by binding to highly specific stem-loop structures. As a result, these systems can be used in combination to visualize two (or more) transcripts in the same cell. However, care must be taken to ensure that insertion of stem-loops into the target transcript does not affect RNA localization. Studies in yeast have indicated that insertion of MS2 stem-lops into the 3’ untranslated region just downstream of the stop codon does not affect transcript localization [67], although it remains necessary to determine empirically the effect of stem-loop insertion on RNA trafficking. A relative disadvantage of these approaches is that they cannot be used to visualize endogenous transcripts. As a result, the transcript of interest must be expressed from a plasmid and therefore may not be subjected to the same transcriptional or post-transcriptional regulation as its cellular counterpart. To circumvent this concern, endogenous transcripts in yeast and mice have been engineered to contain MS2 stem-loops [67,68], although this approach is not practical for many applications.

Despite the limitations and caveats of RNA binding protein-based systems for RNA visualization, MS2-GFP and Bgl-mCherry have provided valuable insight into retroviral RNA trafficking, interactions with host factors, mechanisms underlying genome encapsidation, and the dynamics of particle assembly. Additionally, we have adopted the λ_N_-GFP system to study MMTV RNA trafficking in living cells. We found that MMTV RNA transiently interacts with cellular RNA processing factors, including components of P-bodies. Proteins associated with P-bodies repress the translation of cellular mRNAs and target these transcripts for long-term storage or degradation [69]. Additionally, P-bodies contain components of the RNA-interference pathway, as well as antiretroviral proteins including APOBEC3G [70]. Accordingly, one function of P-bodies may be to restrict retroviral replication. However, proteins associated with P-bodies may also facilitate retroviral replication. For example, the P-body associated helicase Ddx3 is required for nuclear export of unspliced HIV-1 RNA [71]. Furthermore, modulating expression of the P-body associated protein Mov10 interferes with HIV-1 replication [72,73,74], and the Gag proteins of HIV-1 and primate foamy virus-1 recruit Ago2 to the genomic RNA to facilitate viral replication [75]. Our observation that MMTV RNA transiently traffics to P-bodies adds support to the idea that diverse retroviruses interact with P-body proteins to facilitate viral replication. The data presented in this review highlight approaches used to reveal novel insights into retroviral biology by observing the dynamic trafficking and interactions of retroviral RNAs.
